# Evaluating the cerebrospinal fluid tap test with the Hellström iNPH scale for patients with idiopathic normal pressure hydrocephalus

**DOI:** 10.1186/s12987-021-00252-5

**Published:** 2021-04-07

**Authors:** Johanna Rydja, Andreas Eleftheriou, Fredrik Lundin

**Affiliations:** 1 Department of Activity and Health, and Department of Biomedical and Clinical Sciences, Linköping University, 581 85 Linköping, Sweden; 2 Department of Neurology, and Department of Biomedical and Clinical Sciences, Linköping University, 581 85 Linköping, Sweden

**Keywords:** Idiopathic normal pressure hydrocephalus, CSF TT, Outcome, Sensitivity, Specificity

## Abstract

**Background:**

The cerebrospinal fluid tap test (CSF TT) is used for selecting shunt surgery candidates among patients with idiopathic normal pressure hydrocephalus (iNPH). We aimed to evaluate the predictive value of the CSF TT, by using the Hellström iNPH scale for shunted iNPH patients with a standardized method.

**Methods:**

One hundred and sixteen shunt-operated iNPH patients were retrospectively included in this study. The gait and balance domains in the iNPH scale were used as outcome measures for the CSF TT and the total iNPH scale score as the postoperative outcome. A positive response to CSF TT was defined as a change of ≥ 5 points in the gait domain and ≥ 16 points in the balance domain. Differences between CSF TT responders and non-responders, sensitivity, specificity, positive and negative predictive values, accuracy, and correlations between changes from baseline to post CSF TT and from baseline to the postoperative follow-up, were calculated.

**Results:**

In the CSF TT there were 63.8% responders in the gait domain and correspondingly 44.3% in the balance domain. CSF TT responders had a significantly better postoperative outcome in the total scale score (gait P ≤ 0.001, balance P ≤ 0.012) and gait CSF TT responders improved more in gait (P ≤ 0.001) and balance CSF TT responders in balance (P ≤ 0.001). No differences between CSF TT gait or balance responders could be found in neuropsychological or urinary continence assessments postoperatively. The sensitivity and specificity of the CSF TT and the outcome of the total iNPH scale score postoperatively were 68.1% and 52.0% for gait and 47.8% and 68.0% for balance, respectively.

**Conclusions:**

The CSF TT, with the Hellström iNPH scale as the outcome measure, has clear limitations in predicting postoperative results. The gait domain may be used to predict outcomes for gait, but the balance domain is too insensitive.

## Introduction

Idiopathic normal pressure hydrocephalus (iNPH) is a condition that usually has slowly progressive symptoms. In combination with a dilated cerebral ventricular system, the main symptoms are gait disturbance and poor balance, together with cognitive decline and urinary incontinence. The disease is caused by altered cerebrospinal fluid (CSF) dynamics, but the underlying pathophysiology is not fully understood [1]. The only treatment is a shunt insertion to drain fluid from the cerebral ventricular system [2]. In order to achieve an optimal postoperative shunt outcome a correct diagnosis is important. However, diagnosing iNPH is challenging since the symptoms mimic other neurodegenerative conditions and can also occur in combination with other diseases [3].

The cerebrospinal tap test (CSF TT), which involves a lumbar puncture (LP) with removal of 30–50 ml CSF and clinical evaluation before and after the LP, is commonly used to predict patients who could benefit from a shunt insertion [4–7]. The CSF TT has mostly been associated with a high positive predictive value (PPV) and a low negative predictive value (NPV) meaning that potential shunt responders would be missed if the indication for surgery is based on a negative CSF TT [7, 8]. The current international guidelines state that the CSF TT can be a tool with prognostic value [1, 9, 10]. However, the relevance of the CSF TT has been questioned due to its low sensitivity [7, 8, 11]. There is no clear definition of what constitutes a positive CSF TT but an objective improvement in gait has been proposed as the most reliable outcome [12, 13]. The role attributed to the CSF TT in iNPH varies among clinicians from very valuable to a test of limited significance. Standardized evaluation methods of the CSF TT would increase the validity and make comparison between different studies easier [14]. In our center, we use the Hellström iNPH scale [15] to evaluate outcome in the four domains gait, balance, neuropsychology and continence, after surgery. The gait and balance domains are evaluated before and after the CFS TT. The Hellström iNPH scale is a standardized method that has rarely been used as an outcome measure in the evaluation of the CSF TT [8, 16].

## Methods

### Aim and design

The aim of this study was to evaluate the prognostic value of the CSF TT, using the Hellström iNPH scale [15] among shunted iNPH patients, in a large single-center cohort. This is a retrospective study from Linköping University Hospital, Sweden. The participants were consecutively included between January 2016 and December 2019 and data was recorded from the patient´s charts. The study was approved by the Swedish Ethical Review Authority, 2019-02260.

### Participants

**Fig. 1:**
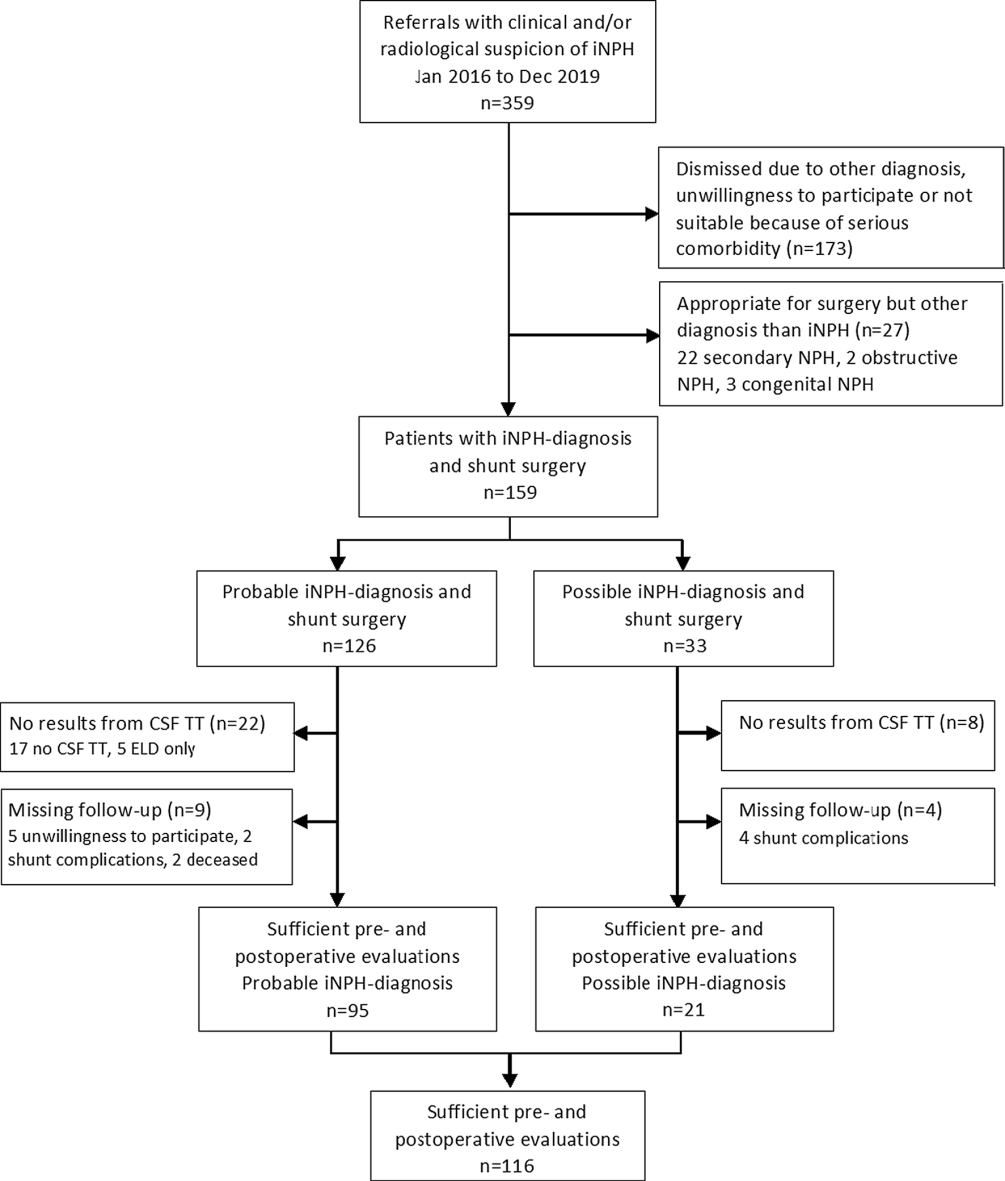
Flow chart of inclusion and exclusion of 116 iNPH patients with results from CSF TT and follow-up evaluation

Patients with a diagnosis of possible and probable iNPH, based on the international guidelines of 2005 [1] and treated with an adjustable valve shunt, were included. All patients had a disturbed gait and balance and additionally an impairment of cognition and/or continence symptoms [1]. Patients were excluded if they had missing data for the CSF TT or the postoperative assessment. Out of 159 iNPH patients, 116 patients (95 patients with probable iNPH and 21 with possible iNPH) were included in the statistical analysis. Forty-three patients were excluded due to following reasons, 25 did not undergo the CSF TT, five had been investigated with external lumbar drainage (ELD) and 13 patients had no result from the follow-up postoperative assessment (six had shunt complications, five had missing data due to unwillingness to participate and two died). One of the deaths was caused by an acute subdural hematoma 2 months after shunt surgery and the other by an intracerebral hematoma 5 days after surgery. A flowchart of inclusion and exclusion is presented in Fig. 1 and the characteristics of the included and non-included participants are shown in Table 1.

**Table 1:** Baseline characteristics of shunted patients who were included and excluded from data analysis

	Patients included probable (n = 95)	Patients included possible (n = 21)	P-value	Patients included total (n = 116)	Patients excluded (n = 43)	P-value
Age, years	76.0 (73.0–80.0)	72.0 (71.0–76.5)	0.02^a^	75.4 ± 6.2	72.8 ± 7.8	NS^a^
Sex, m/f (m %)	51/44 (57.9)	11/10 (52.4)	NS^b^	62/54 (53.4)	29/14 (67.4)	NS^b^
Hypertension, n (%)	60 (63.2)	8 (38.1)	0.04^b^	68 (58.6)	22 (51.2)	NS^b^
Diabetes, n (%)	24 (25.3)	8 (38.1)	NS^b^	32 (27.6)	13 (30.2)	NS^b^
Ischemic heart disease, n (%)	24 (25.3)	4 (19.1)	NS^b^	28 (24.1)	7 (26.3)	NS^b^
Stroke, n (%)	5 (5.3)	6 (28.6)	< 0.01^b^	11 (9.5)	4 (9.3)	NS^b^
MMSE-SR, Scores (0–30)	26.0 (22.8–28.0) n = 94	25.0 (23.0–28.0)	NS^a^	26.0 (23.0–28.0) n = 115	27.0 (23.0–28.0) n = 37	NS^a^
Total iNPH scale score preop (0–100)	53.5 ± 15.7	44.0 ± 20.0	0.02^c^	51.8 ± 16.9	55.7 ± 17.5 n = 34	NS^c^
Volume drained at CSF TT (ml)	48.0 (44.0–50.0) n = 88	50.0 (45.0–50.0) n = 15	NS^a^	48.0 (44.0–50.0) n = 103	47.5 (41.8–50.0) n = 20	NS^a^
Opening Pressure CSF TT (cmH2O)	15.0 (12.0–18.0)	17.8 (15.5–23.0) n = 14	0.01^a^	15.0 (12.5–18.0) n = 109	18.0 (15.0–22.0) n = 23	0.01^a^
Duration baseline to surgery, (days)	120.0 (99.0–157.0)	135.0 (116.0–177.0)	NS^a^	126.0 (102.3–159.0)	133.0 (100.5–171.8) n = 34	NS^a^
Duration surgery to follow-up, (days)	107.0 (90.0–131.0)	97.0 (87.0–120.5)	NS^a^	104.0 (90.0–127.5)	108.0 (93.3–191.8) n = 26	NS^a^
Shunt adjustment before follow-up, n (%)	16 (16.8)	2 (9.5)	NS^b^	18 (15.5)	10 (24.4) n = 41	NS^b^
Shunt revision before follow-up, n (%)	8 (8.4)	3 (14.3)	NS^b^	11 (9.5)	13 (31.7) n = 41	< 0.01^b^

MMSE-SR, Mini Mental State Examination Swedish revision, CSF TT, cerebrospinal fluid tap test

^a^Independent samples Mann–Whitney U test, ^b^Chi square test, ^c^t-test. Values are presented with median and interquartile range (IQR), mean ± SD or as proportions (%). P ≤ 0.05

### Clinical assessments

A specialized team including neurologists, neurosurgeons, neuroradiologists, physiotherapists and occupational therapists assessed the patients preoperatively. The occupational therapist and physiotherapist assessments, accomplished within one week before the CSF TT, included all the measurements for gait, balance, neuropsychology and continence in the Hellström iNPH scale [15]. A neurologist performed the LP, with the patient in a lateral position. A spinal fluid manometer was used to measure the lumbar CSF pressure for 30 s before removal of CSF. The exact amount of removed CSF was noted.

Approximately 3 h after the LP, a physiotherapist reassessed the patients, using the measurements within the gait and balance domains in the iNPH scale [15]. In addition to the standardized assessments, the gait pattern was video-recorded. The neuropsychological and continence domains were not evaluated at this stage after the CSF TT. The patients were offered shunt surgery after a decision was made, based on a clinical judgement, taking all available clinical, radiological and laboratory data into account. No patient was excluded due to a negative CSF TT. The scheduled time for follow-up after surgery was 3 months. At the follow-up the physiotherapist and occupational therapist used the same instruments as preoperatively, including all domains in the iNPH scale [15]. Before follow-up it was checked that the patients had not undergone a recent shunt valve adjustment, otherwise the follow-up was postponed until 3 months later.

### Outcome measures

**Fig. 2:**
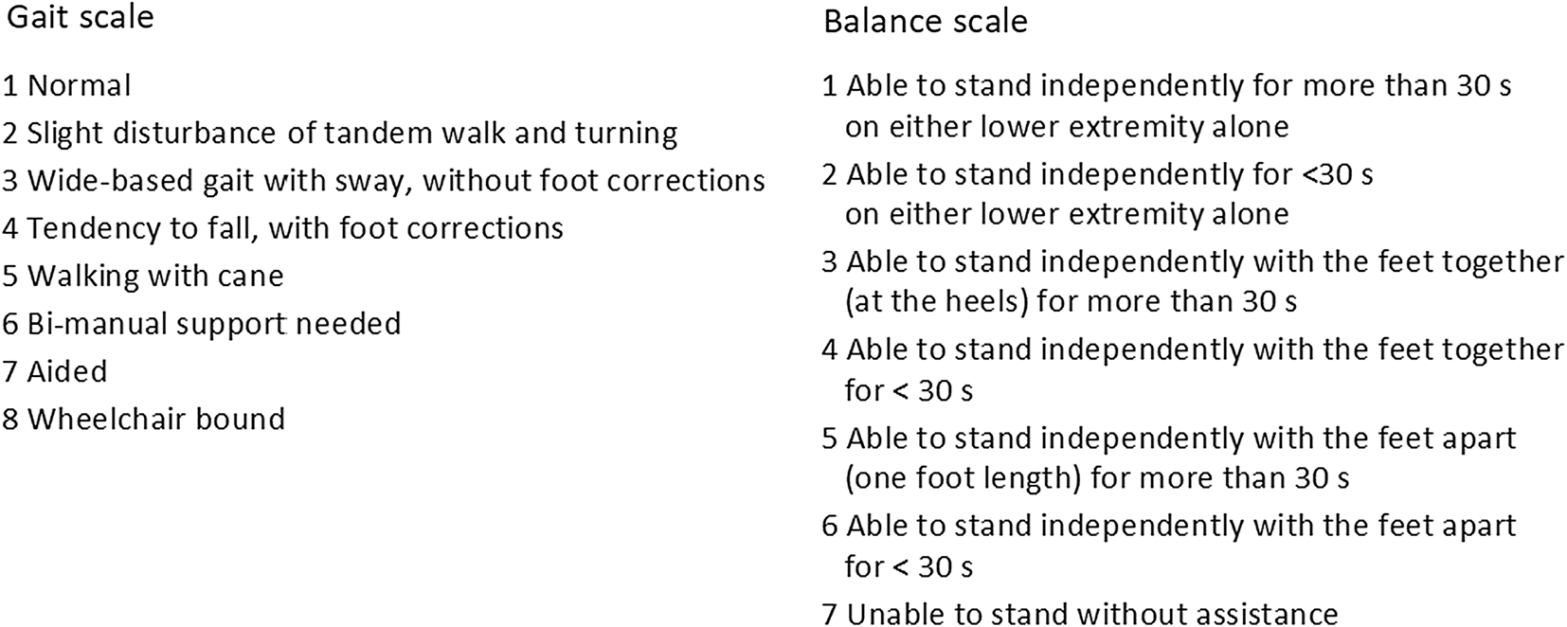
The ordinal grading steps for gait and balance in the Hellström iNPH scale [15]

The Hellström iNPH scale [15] is constructed to cover the symptoms of gait, balance, urinary continence and neuropsychology using ordinal ratings and continuous measures. The gait domain score is calculated from a combination of an eight-grade ordinal gait scale, grading the gait severity (Fig. 2) and the number of steps and time in seconds needed to walk 10 m. Balance is measured with a seven-grade ordinal scale (Fig. 2) and continence with a six-grade ordinal scale. The neuropsychology domain uses four continuous measures. A limit of ≥ 5 points for the total iNPH scale is proposed as an improvement after surgery [15].

According to the instructions by Hellström et al. the results in each domain were converted to a 0–100 score, where 100 represents the performance of an age-matched healthy population. Each step in the ordinal balance scale was converted to 16 or 17 scores in the iNPH scale. A total score was calculated from the converted scores with a double weight from the gait domain and divided by the number of domain scores. If any domain had missing data, the total score was calculated with the available data [15].

### Statistical analysis

Normal distribution was tested with the Kolmogorov–Smirnov test. Normal distributed variables are presented with mean and Standard Deviations (SD), otherwise with median and interquartile range (IQR) or absolute and relative frequencies (%). In Table 2 all values are presented with mean and SD and the different tests used for the comparisons are highlighted according to the normal distributions. For normally-distributed variables the t-test was used otherwise the independent Mann–Whitney U test or Chi square test in comparisons between groups. The related samples Wilcoxon signed rank test was used for comparisons within groups. An improvement of ≥ 5 points for the gait domain and separately at least 16 points in the balance domain (one step in the ordinal balance scale) on the iNPH scale was considered to indicate a positive responder for each of these domains in the CSF TT. Sensitivity, specificity, positive and negative predictive values and accuracy were calculated. The Spearman rank-order test was used to calculate correlation between variables. The level of significance was set to ≤ 0.05. Statistical analysis was carried out using IBM SPSS Statistics version 25.0, (IBM Corp. Armonk, NY).

**Table 2:** Pre- and postoperative values and differences in iNPH scale scores for CSF TT responders and non-responders

	CSF TT Gait responders (n = 74)	Within group differences P value	CSF TT Gait non-responders (n = 42)	Within group differences P value	Between group differences P value	CSF TT Balance responders (n = 51)	Within group differences P value	CSF TT Balance non-responder (n = 64)	Within group differences P value	Between group differences P value
Gait domain scores										
Preop	47.5 ± 19.5		39.5 ± 26.2		0.031^c^	42.4 ± 20.8		46.6 ± 23.6		NS^a^
Postop	71.2 ± 23.7		49.2 ± 24.4		< 0.001^a^	64.9 ± 23.7		61.4 ± 27.9		NS^a^
Diff. preop-postop	23.7 ± 20.4	< 0.001^a^	9.7 ± 16.8	0.001^a^	< 0.001^c^	22.5 ± 21.0	< 0.001^a^	14.9 ± 18.5	< 0.001^a^	NS^c^
10 m (steps, n)^d^										
Preop	25.6 ± 14.0		32.4 ± 21.3		0.039^c^	29.1 ± 20.6		27.1 ± 14.2		NS^c^
Postop	19.5 ± 5.2		23.0 ± 5.9		< 0.001^c^	19.7 ± 4.5		20.9 ± 6.3		NS^c^
Diff. preop-postop	6.6 ± 13.2 n = 73	< 0.001^b^	9.4 ± 20.0 n = 40	< 0.001^b^	NS^c^	9.2 ± 20.0 n = 49	< 0.001^b^	6.2 ± 12.0 n = 63	< 0.001^b^	NS^c^
10 m (time, sec)^d^										
Preop	15.1 ± 6.1		22.0 ± 18.4		NS^c^	17.4 ± 10.9		17.6 ± 13.6		NS^c^
Postop	10.7 ± 4.0		13.9 ± 5.2		< 0.001^c^	11.3 ± 3.6		12.3 ± 5.4		NS^c^
Diff. preop-postop	4.3 ± 5.1 n = 73	< 0.001^b^	8.1 ± 16.5 n = 40	< 0.001^b^	NS^c^	6.0 ± 10.0 n = 49	< 0.001^b^	5.3 ± 11.4 n = 63	< 0.001^b^	NS^c^
Ordinal gait scale scores^d^										
Preop	3 (1–7)		3.5 (3–6)		0.011^c^	3 (2–7)		3 (1–8)		NS^c^
Postop	2 (1–4)		3 (2–4)		< 0.001^c^	2 (1–4)		2 (1–8)		NS^c^
Diff. preop-postop	1 (1–6)	< 0.001^b^	0 (1–4)	< 0.001^b^	NS^c^	1 (− 1–6)	< 0.001^b^	1 (− 1–3)	< 0.001^b^	NS^c^
Balance domain scores										
Preop	65.9 ± 16.4		55.3 ± 23.2		0.006^c^	52.2 ± 19.2		70.2 ± 16.5		< 0.001^c^
Postop	76.2 ± 12.6		65.7 ± 17.8		< 0.001^c^	72.0 ± 14.2		72.8 ± 16.6		NS^c^
Diff. preop-postop	10.3 ± 18.6	< 0.00^b^	10.4 ± 20.6	0.002^b^	NS ^c^	19.8 ± 22.4	< 0.001^b^	2.6 ± 12.0	NS^b^	< 0.00^c^
NP domain scores										
Preop	55.0 ± 18.4		42.5 ± 23.4		0.006^a^	51.7 ± 21.8		50.0 ± 20.6		NS^a^
Postop	60.1 ± 19.5		48.6 ± 22.8		0.008^a^	57.8 ± 21.7		54.9 ± 21.4		NS^a^
Diff. preop-postop	4.9 ± 11.3 n = 69	0.001^a^	4.5 ± 12.6 n = 37	0.037^a^	NS^c^	4.6 ± 12.6 n = 43	0.006^a^	4.6 ± 12.6 n = 60	0.007^a^	NS^c^
Continence domain scores										
Preop	60.7 ± 27.0		55.5 ± 25.8		NS^c^	60.9 ± 23.9		57.7 ± 28.5		NS^c^
Postop	71.6 ± 26.2		71.0 ± 27.0		NS^c^	74.1 ± 23.3	0.001^b^	58.9 ± 28.4		NS^c^
Diff. preop-postop	12.1 ± 27.4 n = 70	< 0.00^b^	15.3 ± 27.7 n = 38	0.002^b^	NS^c^	14.4 ± 25.9 n = 45		11.6 ± 28.3 n = 61	0.003^b^	NS^c^
Total iNPH scale scores										
Preop	55.2 ± 13.3		45.7 ± 20.6		0.010 ^a^	48.9 ± 16.3		54.3 ± 17.0		NS^a^
Postop	70.3 ± 16.5		55.4 ± 16.7		< 0.001^a^	65.8 ± 16.4		63.9 ± 19.3		NS^a^
Diff. preop-posto	15.1 ± 14.5	< 0.001^a^	9.6 ± 11.9	< 0.001^a^	< 0.001^c^	16.8 ± 15.0	< 0.001^a^	9.7 ± 11.3	< 0.001^a^	0.012^c^

CSF TT, Cerebrospinal fluid tap test

CSF TT Gait responders, improvement ≥ 5 points in the gait domain on the iNPH scale

CSF TT Balance responders, improvement ≥ 16 points in the balance domain on the iNPH scale

NP, Neuropsychology

^a^t-test, ^b^Related samples Wilcoxon signed rank test, ^c^Independent samples Mann–Whitney U-test. ^d^subtest within the gait domain. P ≤ 0.05. Values are presented as mean ± SD or median (min–max)

## Results

One hundred and sixteen individuals, 62 male and 54 female with a mean age of 75.4 ± 6.2 years were included in the analysis. Ninety-five patients were classified retrospectively from the patients´ charts by AE and FL as probable iNPH and 21 as possible iNPH according to the international guidelines [1]. Among the 116 individuals, 74 (63.8%) were CSF TT responders with a positive outcome (≥ 5 points) in the gait domain on the iNPH scale. In the balance domain, 51 of 115 individuals, (44.3%), were CSF TT responders (≥ 16 points). Postoperatively, 91 individuals (78.4%) improved ≥ 5 points on the total iNPH scale, with a median improvement of 10 points (IQR: 3.0–20.0). Eighteen patients (15.5%) had at least one shunt valve readjustment, 11 patients (9.5%) had a shunt revision and the median time to follow-up was 104.0 days (IQR: 90.0–127.5). Baseline characteristics and information about the opening lumbar CSF pressure and drained volume in the CSF TT of included and excluded patients are presented in Table 1.

The gait domain CSF TT responders had significantly larger postoperative improvements from baseline in the total iNPH scale scores (gait domain CSF TT responders n = 74; median 13.5, IQR 7.0–20.0; mean 15.1, SD 14.5 versus non-responders n = 42; median 9.5, IQR 0–17.0; mean 9.6, SD 11.9); P ≤ 0.001. The gait domain CSF TT responders improved also significantly more in the gait domain scores postoperatively than the gait domain CSF TT non responders (gait domain CSF TT responders n = 74; median 23.0, IQR 9.8–35.3; mean 23.7, SD 20.4 versus non-responders n = 42; median 7,0 IQR − 3.5–20.0; mean 9.7, SD 16.8); P ≤ 0.001 (Table 2).

The gait domain CSF TT non-responders had significant lower scores at baseline in the gait domain scores (P = 0.031), in the balance domain scores (P = 0.006), in the neuropsychology domain scores (P = 0.006) and in the total iNPH scale scores (P = 0.010) (Table 2).

When analyzing results from the subtests within the gait domain (the 10 m walk test, steps and time and the ordinal gait scale) there were no between-group differences for gait domain CSF TT responders versus gait domain CSF TT non-responders in changes from baseline to the postoperatively follow up. At baseline there were between group differences between the gait domain CSF TT responders and non-responders in the 10 m walk test (number of steps; P = 0.039) and scores in the ordinal gait scale (P = 0.011) (Table 2).

The balance domain CSF TT responders had significantly larger postoperative improvements from baseline in the total iNPH scale scores (balance domain CSF TT responders n = 51; median 15.0, IQR 7.0–21.0; mean 16.8, SD 15.0 versus non-responders n = 64; median 9.0, IQR 3.3–16.5; mean 9.7, SD 11.3); P = 0.012. The balance domain CSF TT responders improved significantly more in the balance domain postoperatively than the balance domain CSF TT non-responders (balance domain CSF TT responders n = 51; median 16.0, IQR 0–34.0; mean 19.8, SD 22.4 versus non-responders n = 64; median 0, IQR 0–0; mean 2.6, SD 12.0); P ≤ 0.001 (Table 2).

The balance domain CSF TT non-responders had significant lower baseline scores in the balance domain (P ≤ 0.001). All other domains as well as for the total iNPH scale scores were equal at baseline (Table 2).

No significant between-group differences among CSF TT responders (gait domain or balance domain) compared to CSF TT non-responders were seen postoperatively in the neuropsychology or continence domains (Table 2).

The sensitivity using the gait domain as outcome in the CSF TT and the total iNPH scale as outcome postoperatively was 68.1% and the PPV 83.8%. The specificity was 52.0% and the NPV was 31.0% with an accuracy of 64.7% (Table 3).

**Table 3:** Sensitivity, specificity, PPV, NPP and accuracy

	Gait domain CSF TT/total iNPH scale postoperatively^a^ N = 116	Balance domain CSF TT/Total iNPH scale postoperatively^a^ N = 115	Gait and Balance domain CSF TT/total iNPH scale postoperatively^a^ N = 116	Gait domain CSF TT/GAIT domain postoperatively^b^ N = 116
Sensitivity	68.1	47.8	29.7	71.8
Specificity	52.0	68.0	76.0	51.6
Positive predictive value	83.8	84.3	81.8	82.4
Negative predictive value	31.0	26.6	23.0	42.9
Accuracy	64.7	52.2	40.0	68.1

Sensitivity, specificity, positive predictive value, negative predictive value and accuracy for the CSF TT with cut off ≥ 5 points in the gait domain or ≥ 16 points in the balance domain or in a combination of both on the iNPH scale

^a^Positive outcome after surgery ≥ 5 points on the total iNPH scale

^b^Positive outcome after surgery ≥ 5 points in the gait domain

The balance domain as an outcome in the CSF TT and the total scale score postoperatively had the sensitivity, 47.8% and a PPV of 84.3%. The specificity was 68.0% and the NPV was 26.6%. The most sensitive output was when using the gait domain as an outcome in the CSF TT and the same outcome postoperatively: sensitivity 71.8%, PPV 82.4%, specificity 51.6% and NPV 42.9% for all ages. When combining both the gait and the balance domain in the CSF TT as outcome the sensitivity decreased to 29.7% (Table 3).

**Fig. 3:**
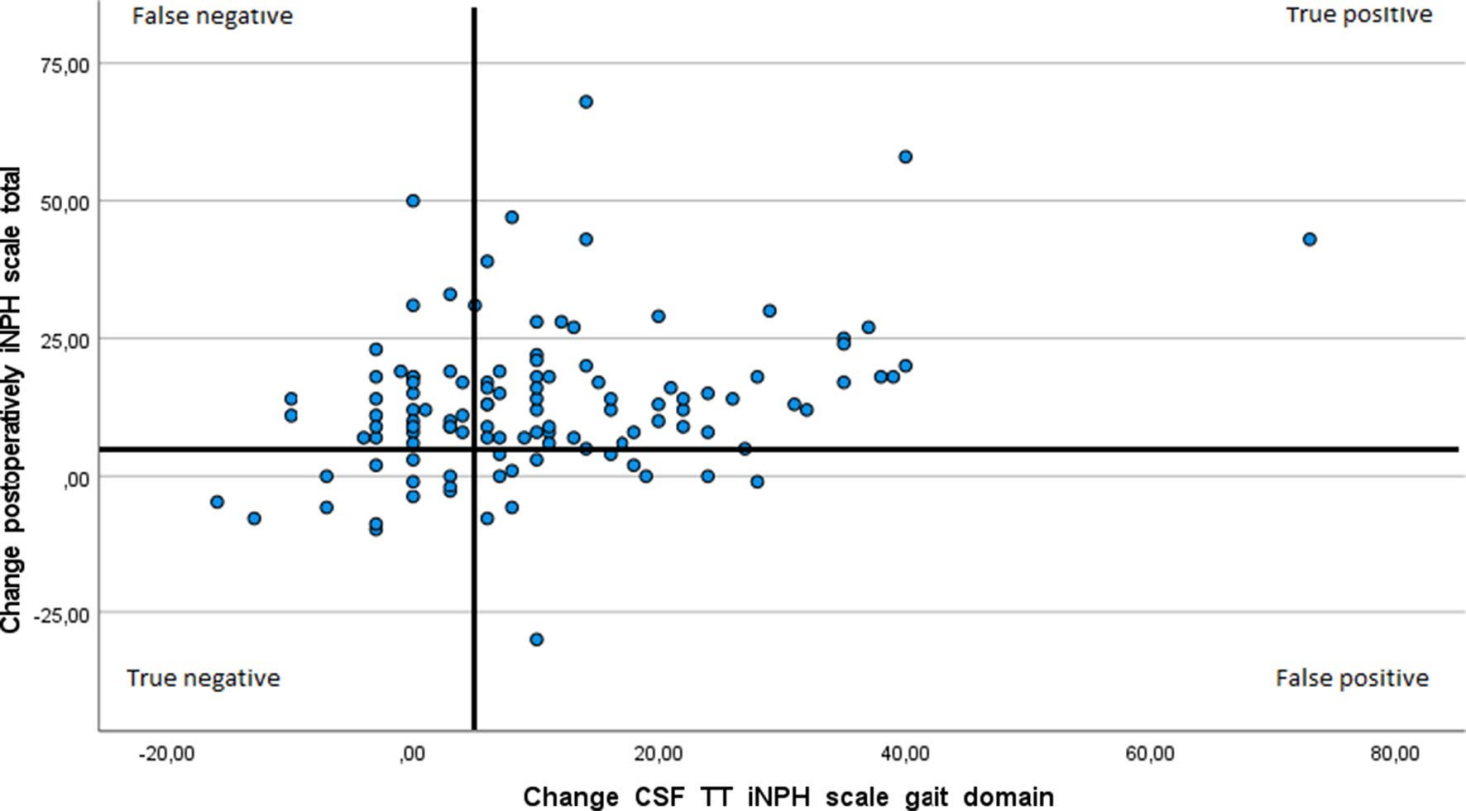
Correlation between changes from baseline at the CSF TT in the gait domain on the iNPH scale and change in the total iNPH scale score postoperatively, Spearman’s rho, r = 0.28, p ≤ 0.01. Distribution of true and false, positive and negative outcomes in the CSF TT with bold lines at the five-point levels of improvement. N = 116

**Fig. 4:**
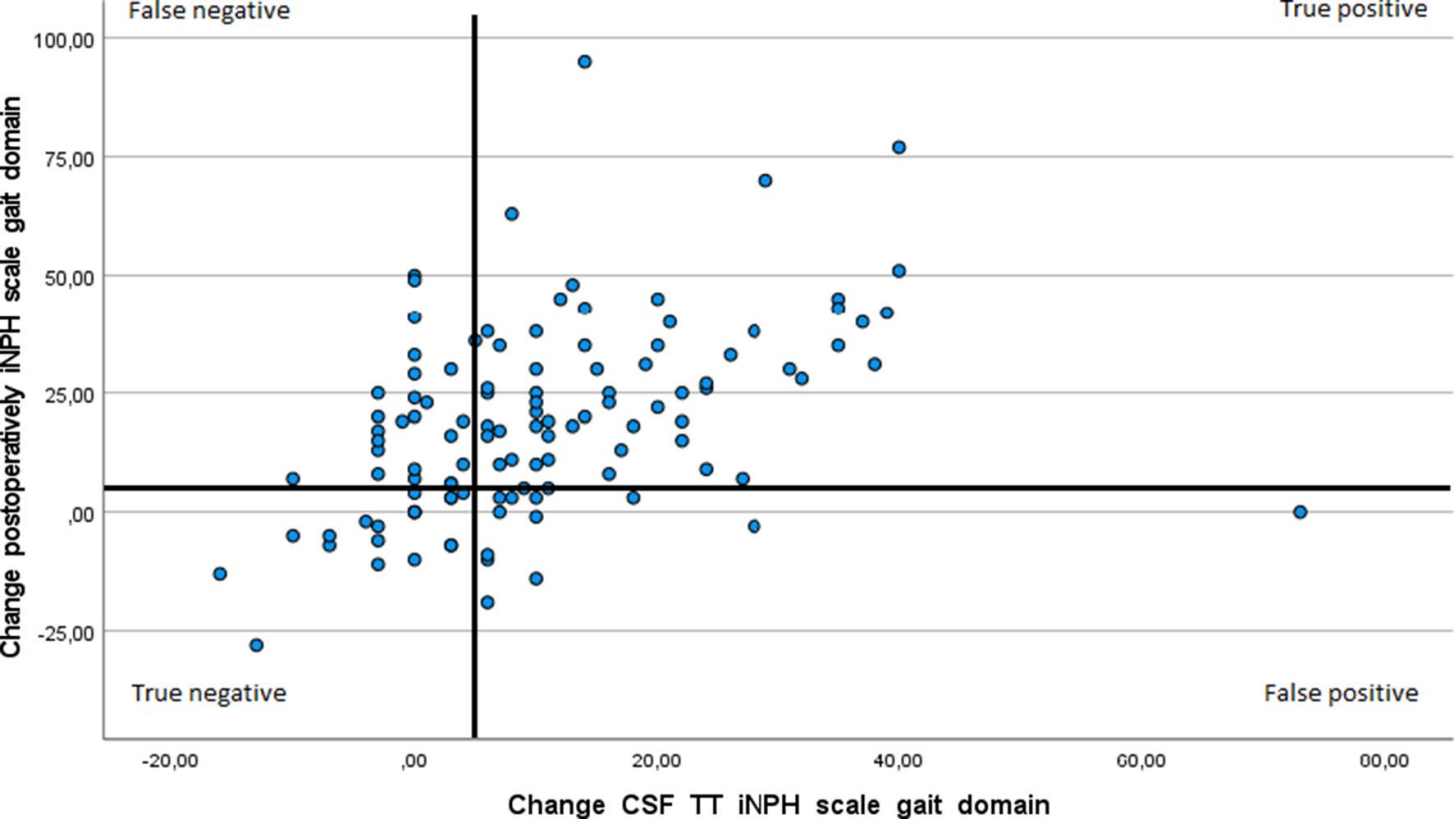
Correlation between changes from baseline at the CSF TT in the gait domain on the iNPH scale and change in the gait domain postoperatively, Spearman’s rho, r = 0.48, p ≤ 0.01. Distribution of true and false, positive and negative outcomes in the CSF TT with bold lines at the five-point levels of improvement. N = 116

The change from baseline in the gait domain score at the CFS TT correlated significantly with change in outcome postoperatively in the total iNPH score, r = 0.28, p ≤ 0.01 as well as with the change in the postoperative gait domain score alone, r = 0.48, p ≤ 0.01. When using the gait domain score as an outcome in the CSF TT and the total iNPH scale scores postoperatively, 62 of 116 (53%) individuals were true positive, 12 (10%) were false positive, 29 (25%) were false negative and 13 (11%) were true negative. With the outcome gait domain scores at the CSF TT and the same outcome (gait domain score) postoperatively, 61 of 116 (53%) were true positive, 13 (11%) were false positive, 24 (21%) were false negative and 18 (16%) were true negative (Figs. 3 and 4).

Of 74 patients with a positive response in the gait domain in the CSF TT, 12 patients had at least one shunt adjustment, of these, 3 patients were negative responders after shunt surgery.

The correlation between changes from baseline in the balance domain at the CSF TT and change in the total iNPH scale score postoperatively was r = 0.38, p ≤ 0.01. Sixty-four of one hundred and fifteen (55.7%) individuals had no change in the balance domain score in the CSF TT.

## Discussion

Predicting a favorable postoperative result from the CSF TT, using the Hellström iNPH scale as an outcome parameter after shunt treatment in iNPH, has clear limitations. Gait domain CSF TT responders improved more in the gait domain and in the total iNPH scale scores from baseline to the postoperative follow-up versus the gait domain CSF TT non-responders. Both gait domain CSF TT responders and gait domain CSF TT non-responders improved significantly in all domains after surgery. The gait domain CSF TT responders performed significantly better in all domains except for continence at baseline (Table 2). The gait domain scores as outcome in the CSF TT and the total iNPH scale scores as postoperative outcome appear to have a moderate sensitivity (68%) and a low specificity (52%) (Table 3). The fact that 56% of the patients (Table 2) had an unchanged balance score in the CSF TT indicates that the balance domain is too insensitive to discriminate small changes.

### Outcome

The difference in outcome measures and timing for assessment, limits the comparability between studies [14]. The choice of outcome measure and defining a cut-off value is essential in all evaluations. Precision, feasibility, clinical relevance and meaningfulness for the patients have to be considered. Gallagher et al. [17] used the Global Rating of Change Scale to evaluate the patient’s perceived improvement to detect minimal clinically important differences in the CSF TT. For example, the cut-off for the Timed up and Go test was a change of 13% and for the Timed up and Go test with an added cognitive task, it was a change of 11%.

The cut-off > 5 points in the total iNPH scale, is suggested by Hellström et al. to identify improvement in the postoperative outcome [15]. The same cut-off level for the gait domain in the CSF TT has not been described. The balance domain cut off level of ≥ 16 points corresponds to the difference between two levels in the ordinal balance scale that should indicate a difference but Hellström et al. [15] were aware of the limitations in separating patients with different levels of symptom severity with the ordinal rating scale used in the balance domain.

We used two different outcomes in the CSF TT, the gait domain and the balance domain in the iNPH scale. The iNPH scale is constructed to cover the most characteristic features in iNPH and to discriminate between patients with variations in symptoms and between patients and healthy individuals. The four domains of the iNPH scale are related but none of the domains could be excluded [15]. In the present study a positive response in the CSF TT in the gait and balance domains were not favourable for the outcome of surgery regarding neuropsychology and continence. This is important to notice since there is sometimes a belief that an improvement in one domain automatically could be transferred into another.

The iNPH scale tolerates missing values but in our study there were few. When analyzing the separate subtests within the gait domain for CSF TT gait domain responders versus CSF TT gait domain non-responders there were somewhat surprisingly no differences in changes from baseline to the postoperative follow up for any of the subtests. In the converted gait domain score there was a significant difference with a larger improvement postoperatively in the CSF TT gait domain responders (Table 2). The construction of the iNPH scale with converted scores and a combination of several subtests in the score is probably one explanation. All subtests and all domains in the iNPH scale increased significantly in both the responder group and the non-responder group postoperatively. It should though be noted that the iNPH scale is validated to evaluate outcomes after surgery [15].

### Prognostic considerations

Our results are consistent with previous research [8, 18, 19] that gait is the most valid variable for detecting changes in the CSF TT. When we used the gait domain, with a cut off level of > 5 points in the CSF TT and the same outcome postoperatively, the sensitivity was 71.8% and the specificity was 51.6% (Table 3). These results are in line with a Japanese multicenter study [19] using the iNPH grading scale with a four-step ordinal scale grading gait, cognition, continence and a total score [20]. Ishikawa et al. [19] used the modified Rankin scale as the postoperative outcome. Eighty percent of the shunted participants were shunt responders, and the sensitivity in the CSF TT, with the total iNPH grading scale score as the outcome, was 71.3% and the specificity was 65.0%. When they used the gait scale score only from the CSF TT (measured 1–2 days after the LP) the sensitivity was 51.3% and the specificity was 85.0%.

In our study, with gait as outcome measure in the CSF TT and the total score as outcome postoperatively, the specificity was lower (52.0%) than in the Japanese study [19]. Even with the high PPV in our study, we had 12 of 116 individuals with a positive response in the CSF TT in the gait domain and a negative outcome after surgery in the total scale score. Deterioration in the other domains postoperatively could be an explanation, but when using the gait domain, as the outcome from both the CSF TT and postoperatively the pattern remains (13 of 116).

This was a retrospective study and a reflection of clinical routine, i.e. there was no blinding and both the patients and the assessors knew the conditions concerning the CSF TT assessment routines. Thus, a placebo effect could be, at least partially, an explanation. Gupta and Lang [21] described a case report of a sham procedure in the CSF TT. In their case walking time decreased corresponding to an improvement of about 30% with both the sham procedure and the large-volume CSF TT.

Another reason for a false positive result from the CSF TT could be a malfunctioning shunt at the follow-up. In our study, we had an accurate registration of shunt adjustments and complications. The shunt should be in a proper condition at the follow up. Three patients with positive outcome of gait in the CSF TT and negative outcome postoperatively hade shunt adjustments before the follow-up.

The relatively long time from baseline to surgery (median 126.0 days) may have negatively influenced the results. Shunt treatment is emphasized to be performed as soon as possible after diagnosis according to the continuous progression of symptoms [22].

### Correlations between CSF TT and postoperative outcomes

In the European iNPH multicenter study, using the Hellström iNPH scale, a correlation between improvement after the CSF TT and outcome post-surgery was only found in the gait domain, with a low correlation (r = 0.22) [8]. In our study the correlation for change after CSF TT and change after surgery in the gait domain was r = 0.48 (Fig. 4). In the same study by Wikkelsø et al. [8], the sensitivity (the total iNPH scale score both in the CSF TT and postoperatively), was 52% and the specificity was 59%. The authors mentioned the suboptimal methodology as a cause of the low predictive value.

Patients within the CSF TT gait responder group in the present study had significantly higher baseline scores than the CSF TT gait non-responders in all iNPH scale domains (except for the continence domain) and in all subtests within the gait domain (Table 2). In previous research it is it has been shown that patients with more severe symptoms have poorer outcome after surgery [22] that also is confirmed in this study.

### The importance of evaluating clinical symptoms and radiology in iNPH

Seventy-eight percent of the operated participants improved on the total iNPH scale score after surgery, which is in line with the 75.0% improvement rate recently reported [23]. The fact that CSF TT was only an adjunct test, and the clinical profile together with the analysis of the radiological characteristics were given priority in the decision of shunt surgery, could be attributed to the positive postoperative outcome among the non-responders.

Due to the high positive predictive value, there are those who support the use of the CSF TT as an additional test to detect shunt candidates. The low negative predictive value emphasizes the importance of not excluding patients from surgery [9, 10, 14]. Even with the standardized methodology in this present study, the diagnostic accuracy of the CSF TT is still limited. A careful evaluation of other clinical symptoms together with a selective radiological assessment and meticulous consideration about other explanatory diagnosis are important aspects to take into account when selecting patients for shunt surgery.

## Strengths and limitations

A limitation of this study is the retrospective design. We included patients over a period of 4 years and there were different examiners during that time. However, strengths of the study are that we used a standardized protocol and prospective inclusion. Using the robust iNPH scale, which tolerates missing values, is a strength and we had few missing data in the included material. The iNPH scale is valid when measuring outcome after surgery and we used the proposed cut-off level. The post CSF TT assessment was measured about three hours after the LP and we used no repetition. Virhammar et al. [24] have reported that improvements in gait can occur with repeated measures, within 24 h after the LP, if previous measurements were negative. Negative outcomes at the time point of three hours, in the present study, could have been falsely negative. Another limitation is the lack of data from the patients who were not selected for surgery.

## Conclusions

Using the gait domain in the Hellström iNPH scale for CSF TT can detect gait outcome after surgery but the negative predictive value is low. The balance domain is too insensitive to use as an assessment of the CSF TT. Clinical examination, accurate radiological assessment and careful consideration of other explanations to cover all aspects of iNPH are important parts in the evaluating process ultimately answering the question who would benefit from shunt surgery.

## Data Availability

The materials and datasets are available from the corresponding author on reasonable request.

## Abbreviations

cmH_2_O: Centimeters of water
CSF: Cerebrospinal fluid
CSF TT: Cerebrospinal fluid tap test
ELD: External lumbar cerebrospinal fluid drainage
iNPH: Idiopathic normal pressure hydrocephalus
IQR: Interquartile range
LP: Lumbar puncture
ml: Milliliter
MMSE-SR: Mini-Mental State Examination, Swedish revision

## References

[1] Relkin N, Marmarou A, Klinge P, Bergsneider M, Black PML (2005). INPH guidelines, part II: Diagnosing idio-pathic normal-pressure hydrocephalus. Neurosurgery..

[2] Bergsneider M, Black PM, Klinge P, Marmarou A, Relkin N. Surgical management of idiopathic normal-pressure hydrocephalus. neurosurgery. 2005;57 suppl_3:S2-29-S2-39. 10.1227/01.NEU.0000168186.45363.4D. 10.1227/01.neu.0000168186.45363.4d 16160427

[3] Malm J, Graff-Radford NR, Ishikawa M, Kristensen B, Leinonen V, Mori E (2013). Influence of comorbidities in idiopathic normal pressure hydrocephalus research and clinical care. A report of the ISHCSF task force on comorbidities in INPH. Fluids Barriers CNS..

[4] Wikkelsö C, Andersson H, Blomstrand C, Lindqvist G, Svendsen P (1986). Predictive value of the cerebrospinal fluid tap-test. Acta Neurol Scand.

[5] Sand T, Bovim G, Grimse R, Myhr G, Helde G, Cappelen J (1994). Idiopathic normal pressure hydrocephalus: the CSF tap-test may predict the clinical response to shunting. Acta Neurol Scand.

[6] Ishikawa M (2004). Clinical guidelines for idiopathic normal pressure hydrocephalus. Neurol Med Chir (Tokyo).

[7] Kahlon B, Sundbärg G, Rehncrona S (2002). Comparison between the lumbar infusion and CSF tap tests to predict outcome after shunt surgery in suspected normal pressure hydrocephalus. J Neurol Neurosurg Psychiatry..

[8] Wikkelsø C, Hellström P, Klinge PM, Tans JTJ (2013). The European iNPH Multicentre Study on the predictive values of resistance to CSF outflow and the CSF Tap Test in patients with idiopathic normal pressure hydrocephalus. J Neurol Neurosurg Psychiatry.

[9] Marmarou A, Bergsneider M, Klinge P, Relkin N, Black PML (2005). INPH guidelines, part III: the value of supplemental prognostic tests for the preoperative assessment of idiopathic normal-pressure hydrocephalus. Neurosurgery..

[10] Mori E, Ishikawa M, Kato T, Kazui H, Miyake H, Miyajima M (2012). Guidelines for management of idiopathic normal pressure hydrocephalus: second. Neurol Med Chir (Tokyo)..

[11] Raneri F, Zella MAS, Di Cristofori A, Zarino B, Pluderi M, Spagnoli D (2017). Supplementary tests in idiopathic normal pressure hydrocephalus: a single-center experience with a combined lumbar infusion test and tap test. World Neurosurg.

[12] Ravdin LD, Katzen HL, Jackson AE, Tsakanikas D, Assuras S, Relkin NR (2008). Features of gait most responsive to tap test in normal pressure hydrocephalus. Clin Neurol Neurosurg.

[13] de Souza RKM, da Rocha SFB, Martins RT, Kowacs PA, Ramina R (2018). Gait in normal pressure hydrocephalus: characteristics and effects of the CSF tap test. Arq Neuropsiquiatr.

[14] Mihalj M, Dolić K, Kolić K, Ledenko V (2016). CSF tap test—Obsolete or appropriate test for predicting shunt responsiveness? A systemic review. J Neurol Sci.

[15] Hellström P, Klinge P, Tans J, Wikkelsø C (2012). A new scale for assessment of severity and outcome in iNPH. Acta Neurol Scand.

[16] Bådagård H, Braun M, Nilsson D, Stridh L, Virhammar J (2020). Negative predictors of shunt surgery outcome in normal pressure hydrocephalus. Acta Neurol Scand.

[17] Gallagher R, Marquez J, Osmotherly P (2019). Clinimetric properties and minimal clinically important differences for a battery of gait, balance, and cognitive examinations for the tap test in idiopathic normal pressure hydrocephalus. Clin Neurosurg.

[18] Damasceno BP, Carelli EF, Honorato DC, Facure JJ (1997). The predictive value of cerebrospinal fluid tap-test in normal pressure hydrocephalus. Arq Neuropsiquiatr.

[19] Ishikawa M, Hashimoto M, Mori E, Kuwana N, Kazui H (2012). The value of the cerebrospinal fluid tap test for predicting shunt effectiveness in idiopathic normal pressure hydrocephalus. Fluids Barriers CNS..

[20] Kubo Y, Kazui H, Yoshida T, Kito Y, Kimura N, Tokunaga H (2007). Validation of grading scale for evaluating symptoms of idiopathic normal-pressure hydrocephalus. Dement Geriatr Cogn Disord.

[21] Gupta A, Lang AE (2011). Potential placebo effect in assessing idiopathic normal pressure hydrocephalus: case report. J Neurosurg.

[22] Andrén K, Wikkelsø C, Tisell M, Hellström P (2014). Natural course of idiopathic normal pressure hydrocephalus. J Neurol Neurosurg Psychiatry.

[23] Giordan E, Palandri G, Lanzino G, Murad MH, Elder BD (2019). Outcomes and complications of different surgical treatments for idiopathic normal pressure hydrocephalus: a systematic review and meta-analysis. J Neurosurg.

[24] Virhammar J, Cesarini KG, Laurell K (2012). The CSF tap test in normal pressure hydrocephalus: evaluation time, reliability and the influence of pain. Eur J Neurol.

